# Extracellular fluid flow and chloride content modulate H^+^ transport by osteoclasts

**DOI:** 10.1186/s12860-015-0066-4

**Published:** 2015-08-15

**Authors:** Priscilla Morethson

**Affiliations:** Department of Physiology and Biophysics, Institute of Biomedical Sciences, University of São Paulo, São Paulo, SP Brazil; Department of Biosciences, Federal University of São Paulo - Unifesp, R. Silva Jardim 136 Vila Mathias, Santos, 11065-201 SP Brazil

**Keywords:** Osteoclast, Intracellular pH regulation, ClC-7, Proton secretion, Bone resorption, Bone extracellular fluid, Fluorescence microscopy, Bis-carboxyethyl-carboxyfluorescein-AM (BCECF-AM)

## Abstract

**Background:**

Bone resorption takes place within the basic multicellular units (BMU), and the surface to be resorbed is isolated from adjacent bone surfaces by a sealing zone between osteoclast membrane and bone matrix, which defines the limits of the resorption lacuna. Considering that the extracellular fluid (ECF) in both BMU and the resorption lacuna can be isolated from its surroundings, I hypothesize that flow and ion composition of the bone ECF in these sites might contribute to the regulation of osteoclast H^+^ secretion. To investigate this hypothesis, I evaluated the H^+^ secretion properties of individual osteoclasts and osteoclast-like cells (OCL-cells) and investigated whether changes in flow or chloride content of the extracellular solution modify the H^+^ secretion properties in vitro.

**Results:**

The results show that 1) osteoclasts are unable to secrete H^+^ and regulate intracellular pH (pH_i_) under continuous flow conditions and exhibit progressive intracellular acidification; 2) the cessation of flow coincides with the onset of H^+^ secretion and subsequent progressive intracellular alkalinization of osteoclasts and OCL-cells; 3) osteoclasts exhibit spontaneous rhythmic oscillations of pH_i_ in non-flowing ECF, 4) pH_i_ oscillations are not abolished by concanamycin, NPPB, or removal of extracellular Na^+^ or Cl^−^; 5) extracellular Cl^−^ removal modifies the pattern of oscillations, by diminishing H^+^ secretion; 6) pH_i_ oscillations are abolished by continuous flowing of ECF over osteoclasts and OCL-cells.

**Conclusions:**

The data suggest, for the first time, that ECF flow and Cl^−^ content have direct effects on osteoclast H^+^ secretion and could be part of a mechanism determining the onset of osteoclast H^+^ secretion required for bone resorption.

**Electronic supplementary material:**

The online version of this article (doi:10.1186/s12860-015-0066-4) contains supplementary material, which is available to authorized users.

## Background

Bone resorption is important for maintaining mineral homeostasis, adapting to functional loading, and healing damaged and fractured sites. The process of bone consumption is regulated by the number of osteoclasts, and relies heavily on the ability of individual osteoclasts to secrete H^+^ through the ruffled border, thus lowering the extracellular pH (pH_o_) at a delimited bone surface.

The first demonstration of an acidic area adjacent to osteoclasts utilized the fluorescent probe acridine orange [1]. Later, it was shown—using pH microelectrodes—that osteoclasts can acidify the contact zone with a culture dish to less than pH 3 within a few minutes [2]. It has been proposed that extracellular acidification is a key step for the dissolution of the apatite-containing mineralized matrix [3, 4] and osteoclast intracellular pH (pH_i_) regulation [5–8]; in addition, H^+^ secretion creates a suitable pH, in the resorption lacuna, for enzymes to degrade the organic matrix [9]. Therefore, bone resorption depends on the expression and activity of H^+^-secreting proteins at the osteoclast ruffled border.

Several mechanisms have been implicated in contributing to the acidification of the resorption lacuna; such as: H^+^ secretion through vacuolar H^+^-ATPase (V-ATPase) [10] and a H^+^-coupled Cl^−^ secretion, by chloride channel 7 (ClC-7) [11]. Furthermore, the Na^+^/H^+^ exchanger, NHE-10 isoform [12], and a H^+^ conductance have been reported to regulate pH_i_ by means of acid secretion [5, 6, 13].

**Fig. 1:**
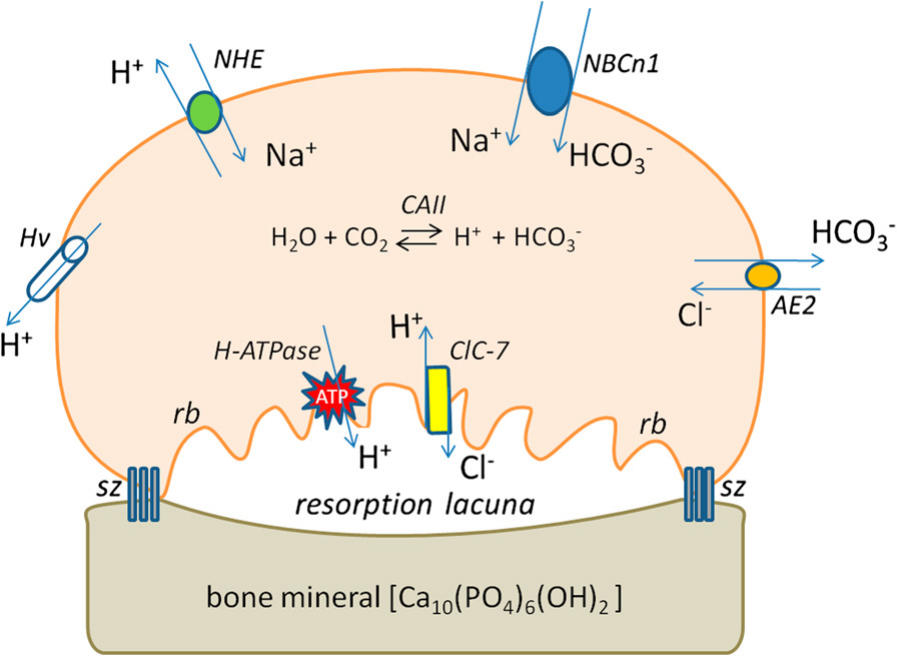
Schematic diagram of proteins involved in acid and base transfer across the osteoclast plasma membrane. RB = ruffled border; SZ = sealing zone; CA = carbonic anhydrase

The movement of acid–base equivalents across the plasma membrane is crucial for pH_i_ regulation [14, 15]. At the osteoclast plasma membrane, the base-transporters include NBCn1 (Na^+^-HCO_3_^−^ cotransporter) [16], and AE2 (Cl^−^/HCO_3_^−^ anion exchanger) [12, 17, 18]; and the acid-transporters include V-ATPase, Na^+^/H^+^ exchanger and the aforementioned H^+^ conductance [5, 6, 13]. In addition to its role in osteoclast pH regulation, H^+^ secretion by the V-ATPase works in parallel with ClC-7 [19], which have been proposed as the key components of cellular machinery for extracellular acidification at the ruffled border (Fig. 1). It should also be noted that pH_i_ regulation is related to the translocation of several ions (as Na^+^ and Cl^−^) across the plasma membrane by specific proteins.

Osteoclast activity and function depends on chemical signaling from cellular and paracrine interactions between osteoclasts and osteoblasts, as well as bone marrow and immune cells, and is regulated by several hormones and cytokines [20]. One of the most important signals for induction of osteoclast differentiation and function is the receptor activator of nuclear kappa-B ligand (RANK-L).

Physical stimuli, for instance functional loading and exercise, also play a role in controlling bone formation, resorption and remodeling. Mechanical loading of bone promotes osteocyte responses to fluid shear stress, which results in changes in the osteocyte activity [21–24] and indirect inhibition of osteoclast formation and bone resorption [25].

Despite the crucial role of osteoclasts in physiological mineral homeostasis and bone adaptive responses, direct effects of flow and ECF ion composition on osteoclast function are otherwise not known. The primary goal of this work is to investigate the effect of extracellular fluid flow rate (5 mL/min versus 0 mL/min) and Cl^−^ content on osteoclast H^+^ secretion. The results show that both extracellular fluid flow and Cl^−^ content modify osteoclast H^+^ transport.

## Results and discussion

### H^+^ secretion after acid load varies among cells in the absence of extracellular solution flow

**Fig. 2:**
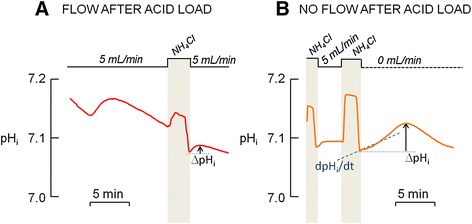
Changes in intracellular pH (pH_i_) of osteoclasts. The pHi of primary osteoclasts labeled with the pH-sensitive dye BCECF-AM (12 μM) was monitored along time in order to evaluate the H^+^ secretion under flow and no-flow conditions after the NH_4_Cl prepulse, which causes an acid load to the cells. Experiments were performed in the absence HCO_3_^−^ to inhibit base transporters. **a.** Flowing standard HEPES-buffered solution (5 mL/min) after acid loading is related to a discrete H^+^ secretion and a small ΔpH_i_. **b**. Non-flowing HEPES-buffered solution (0 mL/min) after acid loading is related to a transient H^+^ secretion and a higher ΔpH_i_ than in flow condition

**Fig. 3:**
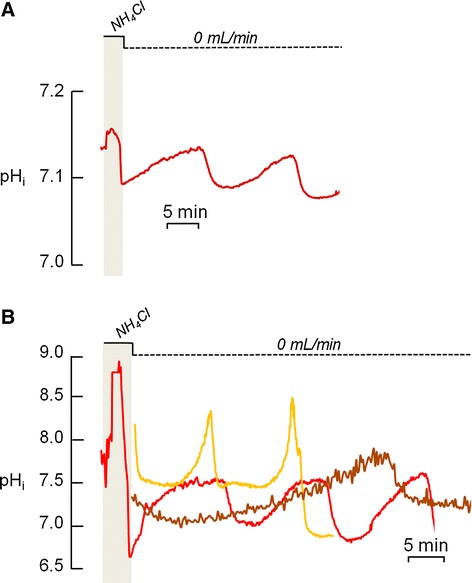
Changes in intracellular pH (pH_i_) of osteoclasts. The pH_i_ of primary osteoclasts labeled with the pH-sensitive dye BCECF-AM (12 μM) was monitored along time in order to evaluate the H^+^ secretion. **a**. Primary osteoclast exhibit rhythmic fluctuations in pH_i_ after the NH_4_Cl prepulse under non-flowing standard HEPES-buffered solution. **b**. Different patterns of pH_i_ oscillations are observed in the primary osteoclasts under non-flowing standard HEPES-buffered solution, with variations in period and amplitude in cycles of acidification and alkalinization, in the presence or absence of NH_4_Cl prepulse. Each line represents one osteoclast

As previously described by Boron and De Weer [26], applying a solution containing NH_4_Cl initially causes an increase in pH_i_. Initially NH_3_ diffuses across the cell membrane and combines with a H^+^ inside the cell; NH_4_^+^ can also move into the cell through K^+^ transporters, bringing extra H^+^, however, this process is slower. The removal of NH_4_Cl solution causes a decrease in pH_i._ The intracellular NH_3_ rushes out the cell, leaving the H^+^ inside. The cell responds to this fall in pH_i_ by activating acid extruding mechanisms. In a set of experiments, I used bis-carboxyethyl-carboxyfluorescein (BCECF-AM) fluorescence to monitor pH_i_ changes using NH_4_Cl prepulse technique. Indeed I was able to monitor pH_i_ changes following acid load (Figs. 2 and 3), in both osteoclasts and OCL-cells. A BCECF-loaded osteoclast is shown in (Additional file 1: Figure S1).

It was observed that osteoclasts do not properly regulate pH_i_ after the NH_4_Cl prepulse in the presence of continuous ECF flow; these cells exhibited a progressive intracellular acidification (n = 6) after discrete recovery from the acid load, which is evidenced by the small ΔpH_i_ (Fig. 2a.) In contrast, when ECF flow was stopped, the osteoclasts secreted H^+^ at different rates (dpH_i_/dt) after the acid load (Fig. 2b), resulting in a higher value of ΔpH_i_ in comparison to that obtained under continuous ECF flow. During the first two minutes following the acid load, the dpH_i_/dt varies from 0.01 to 0.58 pH units/min (n = 27). The values for each osteoclast studied are presented in Additional file 2. The rates of H^+^ secretion do not appear to be related to cell size; however, there is a positive correlation (r = 0.4522, p = 0.0089, n = 27; one-tailed Pearson) between dpH_i_/dt and the magnitude of acidification (i.e. the difference between initial pH_i_ and the lowest pH_i_ recorded after NH_4_Cl solution removal). This correlation was also reported by Ravesloot et al. [6] and may be related to the amount of H^+^ to be transported after the acid load.

### Osteoclasts and OCL-cells exhibit an oscillatory pH_i_ in non-flowing extracellular solution

Under no ECF flow conditions, H^+^ secretion after the acid load causes intracellular alkalinization to a maximal pH_i_ value. Subsequently, the cells initiate a period of spontaneous cellular acidification to a minimal pH_i_ value; then the alkalization restarts. This oscillatory pattern is repeated in regular periods of spontaneous acidification and alkalinization (Fig. 3a). These rhythmic pH_i_ fluctuations were observed in both osteoclasts and OCL-cells, and were observed in 92 % of experiments performed in the absence of ECF flow. Different patterns of pH_i_ oscillation, with different amplitudes and periods, were also observed (Fig. 3b). In osteoclasts, the period of the oscillations—the time interval between two maximum pH_i_ values—ranged from 12 to 45 min (n = 35), and the amplitude of the pH_i_ oscillations—the difference between the maximum and minimum pH_i_ in each cycle—ranged from 0.12 to 1.43 pH units, the lowest and the highest values recorded, respectively. This huge variation in capability of H^+^ secretion among osteoclasts could be due to unequally expression of H^+^-transporting proteins at the plasma membrane, and can be taken as an additional evidence for the osteoclast heterogeneity regarding resorptive machinery already reported by Everts et al. in [27]. Despite the fact that osteoclasts differ from each other in values for period and amplitude of oscillations, one osteoclast analyzed individually exhibits a regular, rhythmic, oscillation, maintaining the range of pH_i_ fluctuation from one cycle to the following and maintaining the period of the oscillations.

The present study is not the first one to report oscillations related to pH_i_ in osteoclasts. In fact, an *in situ* study using microelectrodes to simultaneously measure H^+^ currents and pH in the microenvironment beneath adherent osteoclasts, showed that there were pH fluctuations in that compartment [2]. Despite the methodological differences—extracellular *versus* intracellular measurements—both processes detect pH changes directly related to H^+^ transported by the osteoclast.

### Inhibition of H^+^-transporting proteins does not abolish the pH_i_ oscillation, but the absence of extracellular Cl^−^ modifies its patterns

**Fig. 4:**
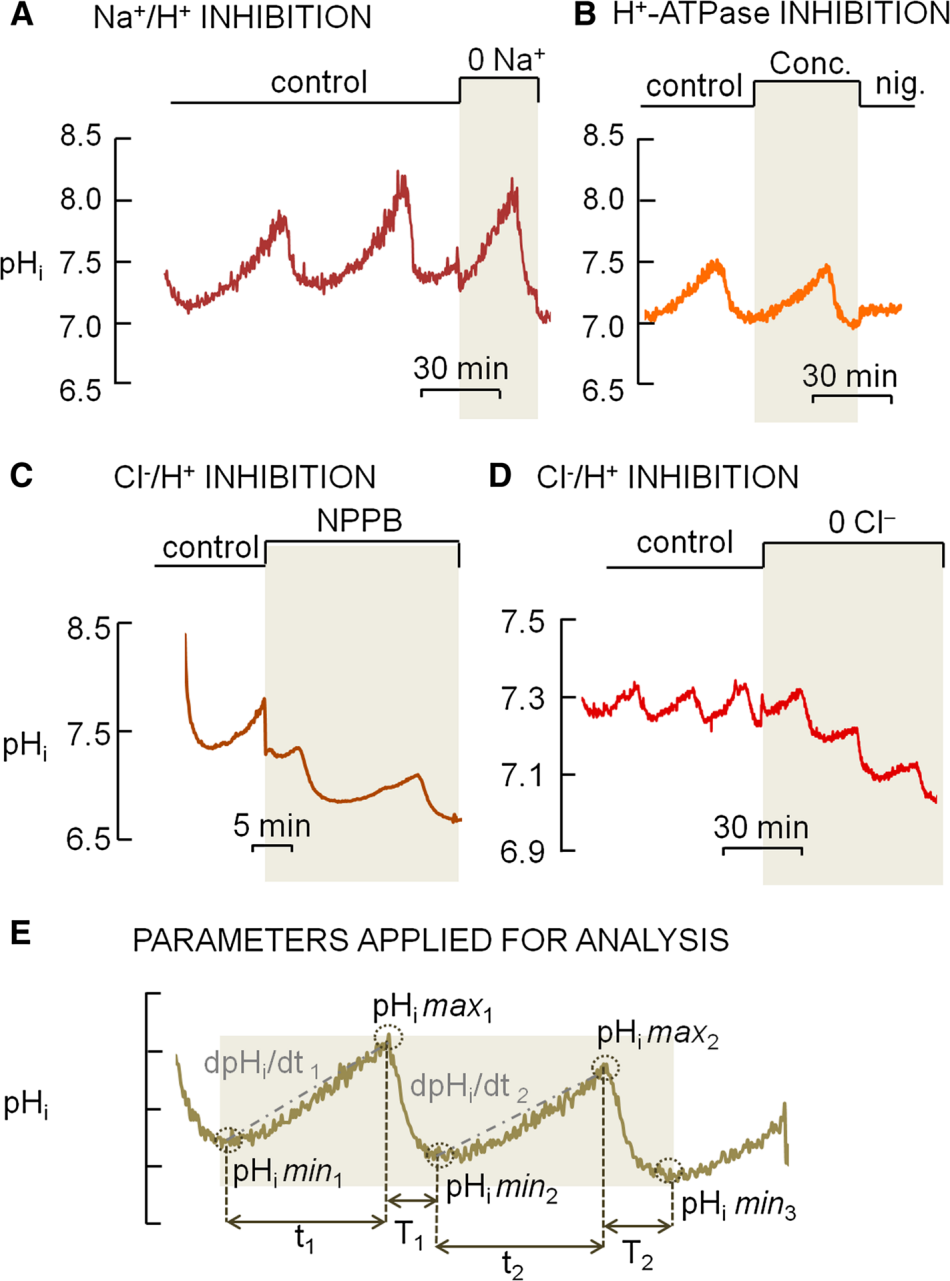
Effect of inhibitors of H^+^-secreting proteins in the oscillating intracellular pH (pH_i_) of primary osteoclasts under non-flowing standard HEPES-buffered solution. **a.** The pH_i_ oscillations were not abolished by applying a zero Na^+^ solution (0 Na^+^), inhibitor of Na^+^/H^+^ exchanger. **b.** The pH_i_ oscillations were not abolished in the presence of concanamycin (Conc.), inhibitor of H^+^-ATPase. (Nig. = nigericin clamps pH_i_ at 7.0.). **c**. The pH_i_ oscillations were not abolished in the presence of NPPB, inhibitor of Clˉ channels. **d**. The pH_i_ oscillations were not abolished by applying a zero Clˉ solution (0 Clˉ), inhibitor of Clˉ transporting proteins; however there is a noticeable and progressive intracellular acidification from one cycle to the next following the removal of extracellular Clˉ (0 Clˉ). **e**. Parameters applied for the analyses of the oscillating pH_i_

The inhibition of the Na^+^/H^+^ exchanger by applying ECF containing zero sodium (0 Na^+^) (n = 5), the inhibition of H^+^-ATPase by concanamycin (n = 3) (Fig. 4a and b) or of H^+^ channels by Zn^2+^ (n = 2) did not disrupt or modify the oscillatory pattern of pH_i_ in osteoclasts. Thus, these H^+^-transporting proteins do not appear to participate in pH regulation by osteoclasts and OCL-cells.

While this may come as a surprise, this is not the first time such an observation has been reported. Grano *et al*. [28] demonstrated that in OCL-cells, Na^+^/H^+^ exchanger inhibition did not completely eliminate the ability of these cells to regulate pH_i_. Another study also showed that total replacement of extracellular Na^+^ had no effect on H^+^ transport in inside-out vesicles derived from the ruffled border of avian osteoclasts [29]. Furthermore, it has been reported that 40 % of osteoclasts containing at least 10 nuclei facilitate pH recovery from acid load through H^+^-ATPase, without any participation from the Na^+^/H^+^ exchanger [30]. While this latter observation is in contrast to our observed results with concanamycin, our data is in accordance with Nordström *et al.* [31], which reported that in the absence of HCO_3_^−^, pH_i_ regulation by H^+^-ATPase is negligible in cells under physiological pH.

The removal of extracellular Cl^−^ (n = 3) or application of NPPB (n = 3), inhibitor of chloride channels, also did not abolish the pH_i_ oscillations (Fig. 4c and d). However, it should be noted that the removal of extracellular Cl^−^ resulted in noticeable difference in the oscillation pattern (n = 3) (Fig. 4d). In control solution, the difference between two maximum values of pH_i_ (pH_i_*max*; Fig. 4e) in two consecutive cycles was −0.03 ± 0.004, whilst in the absence of extracellular Clˉ, the difference between two consecutive values of pH_i_*max* raised to −0.10 ± 0.007, indicating a compromised ability to secrete H^+^. The mean time of intracellular acidification (T; Fig. 4e) was ~6 min under control conditions and was increased to ~9 min in the absence of extracellular Clˉ, which may be related to a decreased ability to secrete H^+^. The mean time of intracellular alkalinization (t; Fig. 4e) was ~15 min under control conditions and was reduced to ~12 min in the absence of extracellular Clˉ, thus shortening the time of H^+^ secretion by 20 %. In control solution, the difference between two minimum values of pH_i_ (pH_i_*min*; Fig. 4e) in two consecutive cycles was −0.01 ± 0.007, whilst in the absence of extracellular Clˉ, the difference between two consecutive values of pH_i_*min* raised to −0.12 ± 0.003, indicating further intracellular acidification. Lastly, the mean rate of intracellular alkalinization (dpH_i_/dt; Fig. 4e) was 0.004 pH units/min under control conditions versus 0.0008 pH unit/min in the absence of extracellular Clˉ, which corresponds to a 5-fold decrease in the H^+^ secretion rate. Since the experiments were performed in the absence of HCO_3_^−^ and because the variations in pH_i_ and dpH_i_/dt are related to H^+^ transport, the reduced ability to secrete H^+^ in the absence of extracellular Cl^−^ could be due to a impaired exchange of external Cl^−^ for internal H^+^ by a Cl^−^/H^+^ exchanger.

The importance of Cl^−^ transporting proteins (channels) in bone resorption emerged when Blair *et al.* [29] demonstrated that in avian osteoclasts the H^+^ secretion through H^+^-ATPase was dependent on an anion conductance, and non-linearly related to the external concentration of Cl^−^. In fact several Cl^−^-transporting proteins are expressed at the osteoclast ruffled border in different species, including ClC-3, ClC-7, and CLIC5 [32–34]. Cl^−^ secretion through these proteins would dissipate the transmembrane potential generated by H^+^-ATPase activity [35].

According to my hypothesis, the absence of extracellular Cl^−^ reduces the exchange of Clˉ by intracellular H^+^, therefore reducing H^+^ secretion, without any participation of H^+^-ATPase. This is supported by data demonstrating that the knockdown of ClC-7 expression by interfering RNA reduces the ability of lysosomal acidification *in vivo,* concluding that ClC-7 is the most important protein for lysosomal acidification [36]. It is also important to consider that bone ECF is not in equilibrium with the bulk extracellular fluid, but instead there is evidence of such a compartimentalization [37]. Higher concentrations of Cl^−^ in bone ECF compared to other extracellular compartments in the body can be taken as evidence that an important physiological role for Cl^−^ exists in bone.

Taken together, the literature and my results provide evidence supporting the following assertion: ClC-7 may itself secrete H^+^ through the ruffled border at the bone-osteoclast interface. This new functional role for ClC-7 presented here is in contrast with the function of charge dissipation, in which Cl^−^ ions would leave the cell in parallel with protons transported by H^+^-ATPase [38]. On the other hand, my hypothesis is in accordance with the first documented biophysical properties of chloride-transporting proteins located at the ruffled border, which was shown to be outward rectifying channels [34]. My proposal of a direct role of ClC-7 in secreting H^+^ at the ruffled border is also in accordance with studies showing that the resorption lacuna of cultured osteoclasts lacking ClC-7 is less acidic [38].

### Fluid flow abolishes pH_i_ oscillations and causes intracellular acidification, but the cessation of flow coincides with the onset of the H^+^ secretion

As previously described in this work, osteoclasts and OCL-cells exhibited spontaneous pH_i_ oscillations in the absence of ECF flow, even in the presence of inhibitors of H^+^ secreting proteins. However, when the ECF was applied at 5 mL/min—value in accordance to the normal intraosseous blood flow rate range reported by Laroche (2002) [39]—the oscillations of pH_i_ were completely abolished in both osteoclasts and OCL-cells. Furthermore, H^+^ secretion after the acid load was absent or brief and not sustained, in both osteoclasts and osteoclast-like cells (OCL-cells) (Fig. 2a).

**Fig. 5:**
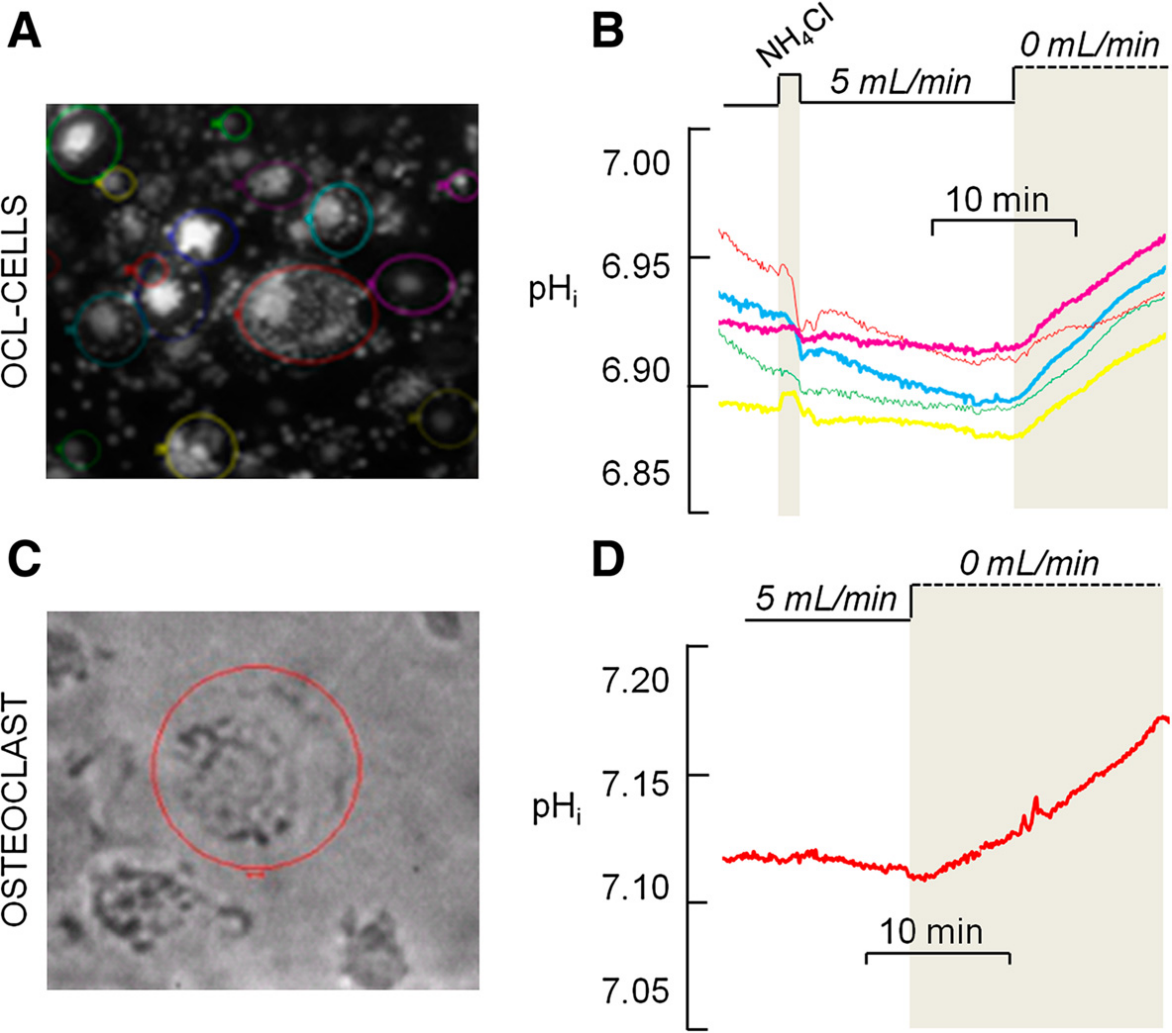
**a**. Emitted fluorescence of BCECF-loaded osteoclast-like cells (OCL-cells) individualized in the area defined by the colored circle line by using the MetaFluor 7.1 software (**b**). pH_i_ of OCL-cells shown in (**a**). Each line represents one OCL-cell. At the time the flow is stopped (dashed line), pH_i_ become progressively more alkaline, thus indicating H^+^ secretion since the standard HEPES-buffered solution does not contain HCO_3_^−^. **c**. Transmitted light image of selected osteoclast. **d**. pH_i_ of the osteoclast shown in **c.** At the time the flow is stopped (dashed line), pH_i_ become progressively more alkaline, thus indicating H^+^ secretion since the standard HEPES-buffered solution does not contain HCO_3_^−^

**Fig. 6:**
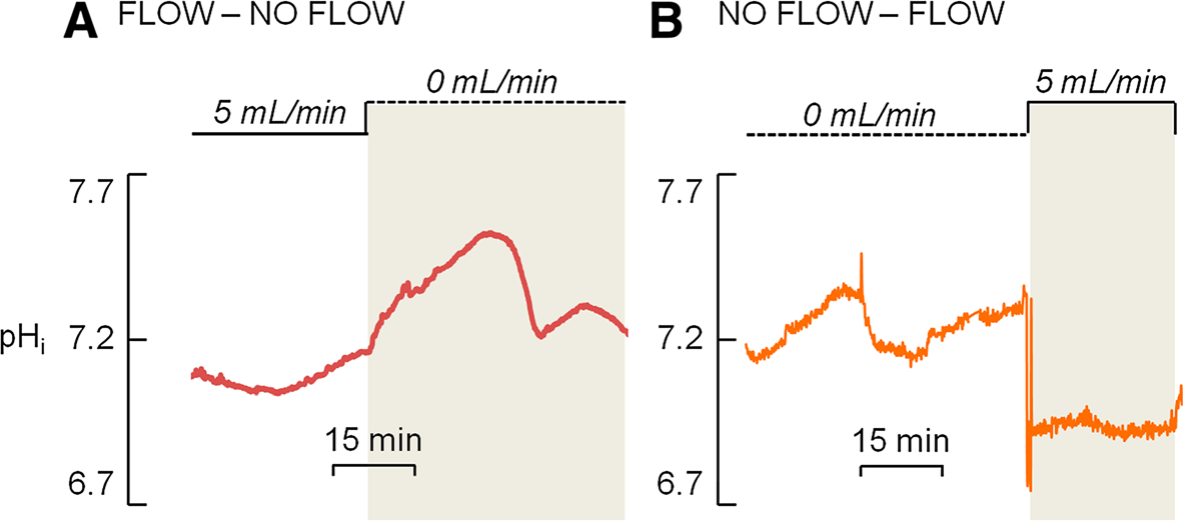
Osteoclast intracellular pH (pH_i_). **a**. The transition from a period of continuous flow (5 mL/min) to a period of non-flowing (0 mL/min) standard HEPES-buffered solution causes pH_i_ oscillation. **b**. Inversely, the transition from a period of non-flowing (0 mL/min) standard HEPES-buffered solution to a period of continuous flow (5 mL/min) abolishes pH_i_ oscillation

In contrast to the results obtained under no flow conditions, with a flow rate of 5 mL/min the cells exhibited progressive intracellular acidification. However, upon the stoppage of extracellular fluid flow H^+^ secretion commences, resulting alkalinization in osteoclasts (n = 6) and OCL cells (n = 20) (Fig. 5) and the previously described oscillatory pattern resumes (Fig. 6a). Furthermore, reapplying flow—after a period of pH_i_ oscillation—disrupts the rhythmic fluctuations in pH_i_ displayed by the osteoclasts (n = 4) and OCL-cells (n = 20) observed under no flow conditions (Fig. 6b). Despite the fact that primary osteoclasts and OCL-cells may have differences concerning phenotype, the similar effect of extracellular fluid flow observed in both cells indicate that the mechanisms related to the effect of fluid in H^+^ secretion properties and pH_i_ regulation are preserved in OCL-cells.

This regulation of H^+^ transport by fluid dynamics of ECF may be relevant during BMU assembly, whereby the canopy over the site to be resorbed isolates local conditions from that in the bulk ECF. In addition, it provides new evidence for a role to intraosseous circulation in regulating the events occuring in the BMU, as highlighted by Parfitt (2000) [40]. The modulation of osteoclast H^+^ secretion by the fluid dynamics here described, along with the regulation of osteoclast function by hypoxia described by Arnett *et al*. (2003) [41], may also serve as regulatory mechanisms for bone resorption in situations where there is stasis or a disruption of the blood supply, such as that occuring in bone fractures or in compression sites during orthodontic treatments.

## Conclusions

In summary, I observed that in the presence of ECF flow, osteoclasts and OCL-cells appear to be unable to secrete H^+^ and the pH_i_ continuously falls. In contrast, when extracellular fluid flow is stopped the cells begin to secrete H^+^ and pH_i_ increases as extracellular pH reduces. The oscillations in osteoclast pH_i_ could be due to the reversion of ion fluxes (i.e. H^+^ and Cl^−^) across the osteoclast plasma membrane. Therefore, it is hypothesized that extracellular Cl^−^ alters the rate of H^+^ secretion by the exchange of Cl^−^ and H^+^ mediated by ClC-7 at the ruffled border. This provides novel evidence for the participation of ClC-7 in osteoclast H^+^ secretion and bone resorption.

My data highlight, for the first time, that flow and Cl^−^ content of the ECF both modulate H^+^ secretion by the osteoclast, independently of H^+^-ATPase, and suggest different and potentially more important roles for ClC-7 in bone resorption than previously recognized. Further studies must be performed in order to better understand these aspects of osteoclast biology.

## Methods

### Isolation of osteoclasts from long bones

**Fig. 7:**
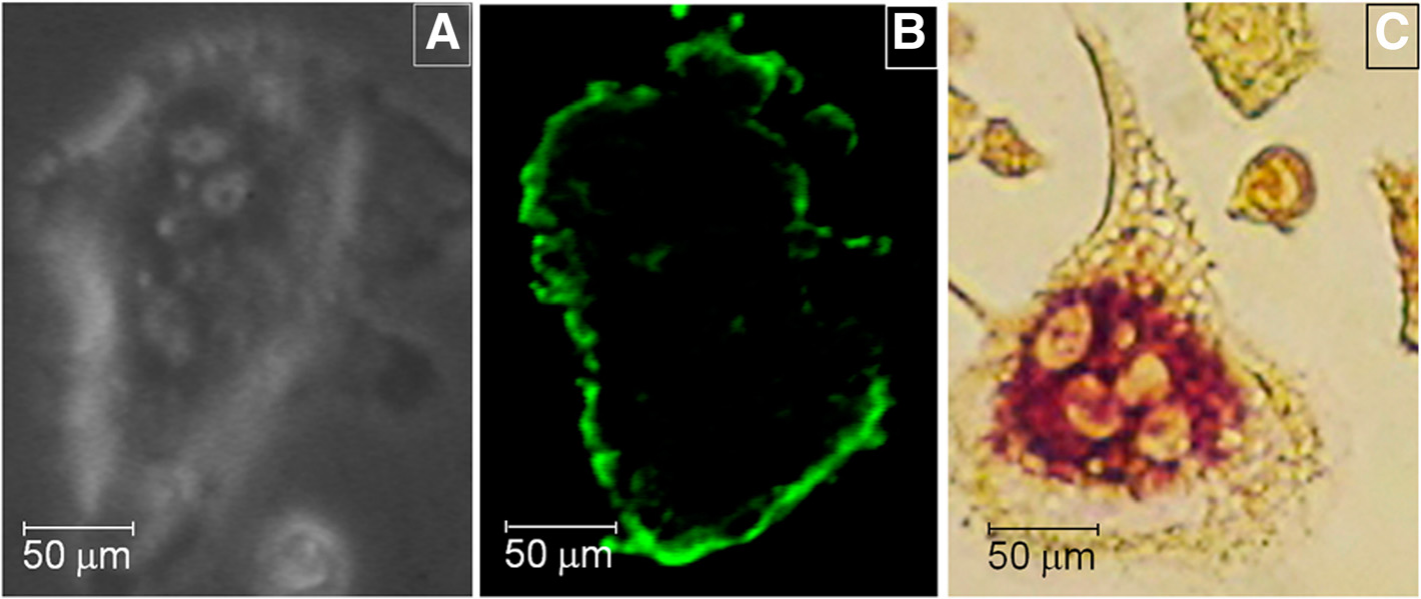
Mature primary osteoclasts were selected based on their morphology, using phase contrast optic system (Olympus IX70; 200 X) (**a**). In some experiments, it was performed further confirmation of the cell phenotype by immunofluorescence using antibody against calcitonin receptor (CTR) (confocal microscope Zeis LSM510; 200 X), a specific marker of osteoclasts (**b**), or by cytochemistry for tartrate resistant acid phosphatase (TRAP) (TRAP kit, Sigma, USA; 200 X) (**c**)

Mature osteoclasts were collected under aseptic conditions from the long bones of newborn Wistar rats. All experimental procedures were performed in accordance with the guidelines of the Standing Committee on Animal Research of the University of São Paulo (Protocol No. 090-35/02). The removed bones were washed with cold α-MEM (Gibco, Grand Island, NE), minced, and cells were detached by repeated pipetting. The debris was allowed to sediment for 30 s, and then the cell suspension was collected. Cells were placed on plastic coverslips at a density of 5 × 10^6^ cells/mL in 300 mOsm/L α-MEM containing 20 IU/L penicillin G, 20 μg/L streptomycin and 0.05 μg/L amphotericin B, 10 % of fetal calf serum and 20 mM N-2- hydroxyethylpiperazine-N'-2-ethanesulfonic acid (HEPES). Cells were kept in a CO_2_ incubator (Lab-Line Instruments, Melrose Park, IL) at 5 % CO_2_, pH 7.4 at 37 °C, for at least two hours before experiments. Mature osteoclasts collected from long bones were selected based on their morphology, using phase contrast microscopy (Olympus IX70, Tokyo, Japan). After selected experiments, further confirmation of the cell phenotype was performed using tartrate resistant acid phosphatase (TRAP) staining kit (Sigma-Aldrich, St. Louis, MO) or by immunocytochemistry using an antibody against the calcitonin receptor (Abcam, Cambridge, UK), a specific marker for osteoclasts (Fig. 7).

### Generation of OCL-cells in vitro

For differentiation of precursor cells in OCL-cells, marrow cells were collected from long bones of 6 to 8 weeks-old Wistar rats. All experimental procedures were performed in accordance with the guidelines of the Standing Committee on Animal Research of the University of São Paulo (Protocol No. 090-35/02). Osteoclast-like cells were generated as described by Arnett *et al*. [41] with modifications; briefly, by using 50 ng/mL of recombinant mouse macrophage colony stimulating factor (rmM-CSF) (R&D Systems, Minneapolis, MN) and 10 ng/mL of recombinant mouse receptor activator of nuclear factor kappa B ligand (rmRANK-L) (R&D Systems). Cells were maintained in 300 mOsm/L α-MEM containing 20 IU/L penicillin G, 20 μg/L streptomycin and 0.05 μg/L of amphotericin B, 10 % of fetal calf serum and 20 mM HEPES on plastic coverslips, in a CO_2_ incubator (Lab-Line Instruments, India) at 5 % CO_2_, pH 7.4 at 37 °C, for up to 7 days. The culture medium was changed every 3 or 4 days.

### Experimental solutions and chemicals

All pH_i_ experiments were performed in the absence of NaHCO_3_, in order to avoid activity of proteins involved in base transport (i.e. Na^+^-HCO_3_^−^ cotransporters). The composition of experimental solutions is shown as additional data. The experimental solutions were maintained at 37 °C regardless of flow conditions.

Concanamycin (Sigma-Aldrich, St. Louis, MO), the H^+^-ATPase inhibitor, was used at a final concentration of 100 mM [5]. NPPB [5-Nitro-2-(3-phenylpropylamino) benzoic acid] (Tocris Bioscience, Bristol, UK), the chloride channel inhibitor, was used at a final concentration of 100 μM [42]. ZnCl_2_ (Sigma-Aldrich, St. Louis, MO), the proton channel inhibitor, was used at a final concentration of 100 μM [5].

### pH_i_ records

The changes in cellular pH_i_ were monitored by measuring the change in 12 μM BCECF-AM (Molecular Probes, Eugene, OR) fluorescence on an Olympus IX70 microscope. The emitted fluorescence after excitation at 490 nm (EF 490 nm) is related to pH_i_, and that emitted after excitation at 440 nm is insensitive to pH_i_, but indicates dye concentration and dye loss by photobleaching during fluorescence records. The pH_i_ was analyzed in the presence or absence of inhibitors of H^+^ ATPase, proton channels and ClC-7; in the presence or absence of extracellular Na^+^ or Cl^−^, in order to inhibit Na^+^ and Cl^−^-dependent mechanisms, respectively; and in the presence (5 mL/min) or absence (0 mL/min) of extracellular fluid flow. The flow and/or exchange of the ECF were controlled using syringe pump (Warner Instruments, Hamden, CT).

I also investigated the effect of acid loading the osteoclasts by exposing the cells to a solution of 20 mM NH_4_Cl for 1–2 min [26]. The entry of NH_3_ into the cell causes a transient alkalinization of pH_i_ due to the reaction of NH_3_ with intracellular H^+^ forming the ion NH_4_^+^; subsequently, the entry of NH_4_ leads to intracellular acidification. The replacement of NH_4_Cl solution for a standard HEPES-buffered solution (pH 7.4, at 37 °C) causes the pH_i_ to fall abruptly. The osteoclasts ability to secrete H^+^ after the acid load was investigated over time in the presence or absence of ECF flow. The rates of H^+^ secretion were calculated as the linear regression—dpHi/dt—for the two first minutes after the lowest pH_i_ value induced by the NH_4_Cl prepulse was obtained, or for the entire period in the analysis of pH_i_ oscillations.

### Data analyzes

The ratiometric approach was used to generate a parameter related to pH rather than dye concentration [14]. Briefly, the emitted light (above 510 nm) after excitation at 490 nm (EF490) and 440 nm (EF440) was recorded using the MetaFluor 7.1 software. The emitted fluorescence after excitation at 490 nm is sensitive to both dye concentration and pH_i_, and the emitted fluorescence after excitation at 440 nm (isosbestic wavelength) is not sensitive to changes in pH_i_. The fluorescence ratio values (R = EF490/EFI440) were converted to pH_i_ using a calibration technique involving the use of high-[K^+^]/nigericin (10 μM) Invitrogen, Waltham, MA) as previously described [6]. Detailed calibration was performed on a set of cells and a single-point calibration was routinely performed at the end of all the pH_i_ records. Representative pH_i_ values of individual osteoclasts and OCL during the course of the experiments were plotted *versus* time.

## Additional files

Additional file 1: Figure S1. BCECF-loaded primary osteoclast on confocal microscope Zeiss LSM 510 (200 X). The mature osteoclast was extracted from 2 days-old Wistar rat and incubated with the pH-sensitive dye BCECF-AM (12 μM) for 10 min at 37°C. **A**. Transmitted light image. **B**. Fluorescence image of BCECF trapped in the cytosol. Note that nuclei are not fluorescent. **C**. Merged image of A. and B. (PNG 260 kb) Additional file 2: Table S1. Rates of H^+^ secretion (dpH_i_/dt) for individual osteoclasts. **Table S2** Composition of experimental solutions. Values are expressed in mM. (DOCX 15 kb)
