# Supplementary figures and images for: Extracellular fluid flow and chloride content modulate H+ transport by osteoclasts
Source: BMC Cell Biol. 2015 Aug 15;16:20. doi: 10.1186/s12860-015-0066-4 (PMC4536797; doi:10.1186/s12860-015-0066-4)

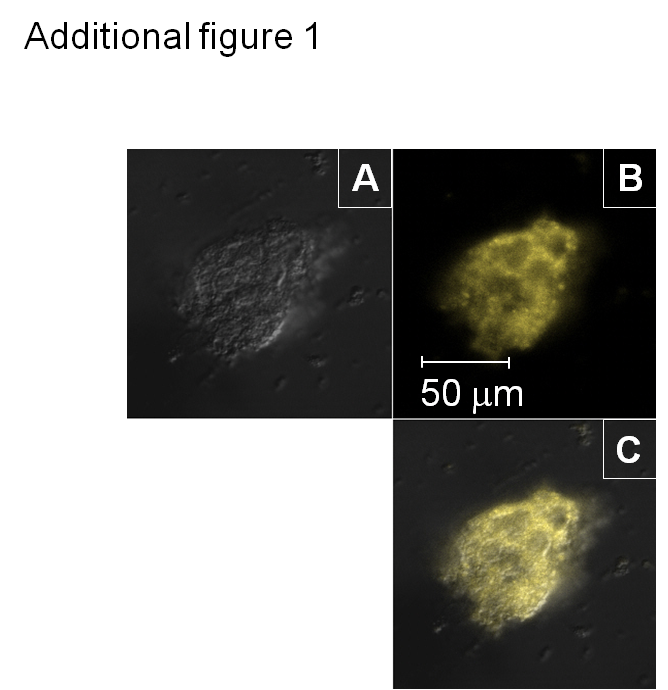

Supplement: Additional file 1: Figure S1. — BCECF-loaded primary osteoclast on confocal microscope Zeiss LSM 510 (200 X). The mature osteoclast was extracted from 2 days-old Wistar rat and incubated with the pH-sensitive dye BCECF-AM (12 μM) for 10 min at 37°C. A. Transmitted light image. B. Fluorescence image of BCECF trapped in the cytosol. Note that nuclei are not fluorescent. C. Merged image of A. and B. (PNG 260 kb) [file 12860_2015_66_MOESM1_ESM.png]
