# Supplementary material for: Extracellular fluid flow and chloride content modulate H+ transport by osteoclasts
Source: BMC Cell Biol. 2015 Aug 15;16:20. doi: 10.1186/s12860-015-0066-4 (PMC4536797; doi:10.1186/s12860-015-0066-4)
Supplement: Additional file 2: Table S1. — Rates of H+ secretion (dpHi/dt) for individual osteoclasts. Table S2 Composition of experimental solutions. Values are expressed in mM. (DOCX 15 kb) [file 12860_2015_66_MOESM2_ESM.docx]

SUPPLEMENTAL DATA

**Table S1.** Rates of H^+^ secretion (dpH_i_/dt) for individual osteoclasts.

| **Initial pH_i_ – Lowest pH_i_ after acid load** | **dpH_i_/dt** |
| --- | --- |
| 1.35 | 0.56 |
| 0.81 | 0.27 |
| 0.41 | 0.57 |
| 0.83 | 0.27 |
| 1.03 | 0.25 |
| 0.80 | 0.58 |
| 0.04 | 0.22 |
| 0.88 | 0.13 |
| 0.59 | 0.01 |
| 0.59 | 0.04 |
| 1.31 | 0.38 |
| 0.84 | 0.29 |
| 1.03 | 0.23 |
| 0.50 | 0.08 |
| 1.32 | 0.57 |
| -0.04 | 0.14 |
| 1.35 | 0.08 |
| 1.42 | 0.17 |
| 0.63 | 0.07 |
| 0.48 | 0.10 |
| 0.57 | 0.04 |
| 0.22 | 0.02 |
| 0.24 | 0.01 |
| 0.84 | 0.05 |
| 0.33 | 0.02 |
| -0.01 | 0.05 |
| 0.46 | 0.09 |

Values of dpH_i_/dt are correlated to the magnitude of acid load, i.e. the difference between initial pH_i_ and the lowest pH_i_ recorded after the acid load.

**Table S2**. Composition of experimental solutions. Values are expressed in mM.

| **Chemicals** | **Control** | **NH_4_Cl** | **BCECF-AM** | **Nigericin** |
| --- | --- | --- | --- | --- |
| **NaCl** | 138 | 140 | 138 | 20 |
| **KCl** | 5 | 5 | 5 | 130 |
| **CaCL_2_** | 1.8 | 1.8 | 1.8 | 1 |
| **MgCl_2_** | - | - | - | 1 |
| **MgSO_4_** | 0.81 | 0.81 | 0.81 | - |
| **NaH_2_PO_4_** | 0.9 | 0.9 | 0.9 | - |
| **HEPES** | 10 | 10 | 10 | 5 |
| **Glucose** | 5 | 5 | 5 | - |
| **NaOH** | 8 | 8 | 8 | - |
| **NH_4_Cl** | - | 20 | - | - |
| **Nigericin** | - | - | - | 0.01 |
| **NaHCO_3_** | - | - | - | - |
| **BCECF-AM** | - | - | 0.012 | - |
| **pH (solution)** | 7.4 | 8.0 | 7.4 | 9.0-5.0 |
